# Chronic folate deficiency induces glucose and lipid metabolism disorders and subsequent cognitive dysfunction in mice

**DOI:** 10.1371/journal.pone.0202910

**Published:** 2018-08-28

**Authors:** Mei Zhao, Man Man Yuan, Li Yuan, Li Li Huang, Jian Hong Liao, Xiao Ling Yu, Chang Su, Yuan Hua Chen, Yu Ying Yang, Huan Yu, De Xiang Xu

**Affiliations:** 1 School of Nursing, Anhui Medical University, Hefei, China; 2 Anhui Provincial Key Laboratory of Population Health & Aristogenics, Hefei, China; 3 Department of Histology and Embryology, Anhui Medical University, Hefei, China; 4 Department of Toxicology, Anhui Medical University, Hefei, China; University of Hawai'i at Manoa College of Tropical Agriculture and Human Resources, UNITED STATES

## Abstract

Previous studies have shown that folate levels were decreased in patients with type 2 diabetes (T2D) and further lowered in T2D patients with cognitive impairment. However, whether folate deficiency could cause T2D and subsequent cognitive dysfunction is still unknown. The present study aimed to explore the effects of chronic folate deficiency (CFD) on glucose and lipid metabolism and cognitive function in mice. Seven-week-old mice were fed with either a CFD or control diet for 25 weeks. Serum folate was significantly reduced, whereas serum total homocysteine was significantly increased in the CFD group. Moreover, CFD induced obesity after a 6-week diet treatment, glucose intolerance and insulin resistance after a 16-week-diet treatment. In addition, CFD reduced the hepatic p-Akt/Akt ratio in response to acute insulin administration. Moreover, CFD increased serum triglyceride levels, upregulated hepatic *Acc1* and *Fasn* mRNA expression, and downregulated hepatic *Cd36* and *ApoB* mRNA expression. After a 24-week diet treatment, CFD induced anxiety-related activities and impairment of spatial learning and memory performance. This study demonstrates that folate deficiency could induce obesity, glucose and lipid metabolism disorders and subsequent cognitive dysfunction.

## Introduction

Type 2 diabetes (T2D) is a chronic and progressive metabolic disorder characterized by hyperglycemia and insulin insensitivity. Approximately 415 million people aged 20–79 years were estimated to have diabetes worldwide in 2015 and the number was predicted to rise to 642 million by 2040. The global health-care expenditure on diabetes was about 673 billion US dollars [1]. People with T2D have higher risk of dementia than those without T2D [2]. Therefore, it is important to identify the potential risk factors of T2D. Subjects with T2D had significantly reduced erythrocyte folate levels compared with nondiabetic subjects [2–3]. A case-control study showed serum folate levels were about 3-fold lower and serum homocysteine levels were significantly higher in patients with T2D compared with healthy controls [4]. Moreover, patients with both T2D and mild cognitive impairment had significantly lower levels of folate compared with patients with T2D and without mild cognitive impairment [5]. Although folate levels were decreased in patients with T2D and further lowered in patients with T2D with cognitive impairment, it is still unknown whether folate deficiency could cause T2D and subsequent cognitive dysfunction.

Folate is an essential vitamin that serves as a source of single carbon units in methionine/homocysteine cycle by supplying 5-methyltetrahydrofolate for the methylation of homocysteine back into methionine [4,6]. Therefore, decreased methionine and increased homocysteine may be a secondary consequence of folate deficiency [7]. Decreased methionine can induce hepatic lipid accumulation by downregulating sterol regulatory element-binding protein (*Srebp1*) mRNA and upregulated the expression of acetyl Coenzyme A carboxylase 1 (*Acc1*) and fatty acid synthase (*Fasn*) mRNA which involved in hepatic lipid synthesis [8]. Liver steatosis is a major risk factor of insulin resistance, a risk factor for the development of T2D [9]. Thus, it was speculated that folate deficiency could induce glucose and lipid metabolism disorders.

Folate deficiency is also a risk factor for the cognitive dysfunction associated with aging [10]. Previous investigations have combined folate deficiency with other vitamin deficiencies in an Alzheimer’s disease mouse model and an apolipoprotein E mouse model [11–12]. Other researchers reported that the vitamin deficiency exacerbated the cognitive impairment [11,13]. A recent study showed that folate deficiency impaired cognition and attention during the nesting test in Ts65Dn mice [14]. However, these studies cannot determine the role of folate in cognitive function due to other B vitamins that were also deficient in the diet. So far, few studies have investigated the effects of folate deficiency on the behavior of mouse models.

Previous studies showed that folate deficiency impaired cognitive function through alterations in the protein homocysteinylation [6], methylation status and oxidative stress [15]. However, the mechanism is not fully understood. On the other hand, patients with T2D are prone to develop cognitive dysfunction [16–17]. Collectively, it was hypothesized that folate deficiency might cause glucose metabolism disorders and subsequent cognitive dysfunction, which may be related to insulin resistance.

The aim of the present study is to investigate the effects of chronic folate deficiency (CFD) on glucose and lipid metabolism and subsequent cognitive function in mice. Our findings suggested that CFD induced obesity, hypertriglyceridemia, disturbance of hepatic lipid-related gene regulation, glucose intolerance and insulin resistance. Subsequently, CFD led to anxiety-related activities and impairment of spatial learning and memory performance, which might be related to CFD-induced insulin resistance.

## Materials and methods

### Animals and treatments

Institute of Cancer Research (ICR) mice have been widely used and growing number of researches were performed in ICR mice aimed to establish metabolic disease model [18–22]. Female ICR (6 weeks old; 20-24g) mice were purchased from Beijing Vital River whose foundation colonies were all introduced from Charles River Laboratories, Inc. (Wilmington, MA, USA). Mice were *ad lib* to water and food and housed on a 12-h light/dark cycle (lights on at 7:00 a.m.) in a controlled temperature (20–25°C) and humidity (50 ± 5%) environment. Mice were fed with standard animal chow for 7 days to adapt to the environment. Twenty mice were randomly divided into two groups of control and CFD (n = 10 per group). Mice were fed with a standard animal chow (2 mg/kg folic acid) or folate deficient diet (0 mg/kg folic acid, with 1% succinylsulfathiazole to suppress microbial folate synthesis) for 25 weeks. All diets were purchased from TROPHIC Animal Feed High-tech Co Ltd (Nantong, Jiangsu, China). The composition of diets was shown in **S1 Table.** Percentage distribution of calories was shown in **S2 Table**.

Mice were monitored at least twice per day and weighed weekly. At the 16th week after diet treatment, it was observed that the water intake was increased and the sawdust bedding was wetter in the CFD group which may be related to impaired glucose homeostasis. Thereafter, intraperitoneal glucose tolerance (IPGTT) and intraperitoneal insulin tolerance tests (ITT) were performed at the 16th and 17th week. At the 24th week after diet treatment, persistent repetitive behaviors, including jumping and backward somersaulting were observed in the CFD group. Thus, open field, elevated plus maze and Morris water maze were conducted to test cognitive function. The day after all tests, blood glucose levels were measured with a glucometer (Roche Accu-Chek Inform) after an overnight fast (23:00–7:00). Thereafter, six mice of either group were i.p. injected with recombinant insulin (1.0 U/kg) or saline, and sacrificed 5 minutes after insulin injection. Blood was rapidly removed by cardiac puncture and centrifuged (4°C, 3000r/min, 15 min) and serum was stored at −80°C for biochemical parameters. The liver, abdominal fat tissues were collected and were either frozen immediately in liquid nitrogen for real time PCR and immunoblot or fixed in 4% paraformaldehyde for histology.

This study was carried out in strict accordance with the recommendations in the Guide for the Care and Use of Laboratory Animals of the National Institutes of Health. All animals were sacrificed by intraperitoneal injection phenobarbital sodium (50 mg/kg). The protocol was approved by the Institutional Animal Care and Use Committee of Anhui Medical University (Protocol Number: LLSC20140088).

### Intraperitoneal glucose tolerance and intraperitoneal insulin tolerance tests

IPGTT was performed after an overnight fast (20:00–8:00) at the 16th week (n = 10 per group). For IPGTT, glucose (2.0 g/kg) was i.p. injected and blood glucose was drawn from the tail before the glucose load (0 min time point) and at 15, 30, 60, and 120 min thereafter. After a week, intraperitoneal insulin tolerance tests (ITT) was performed after 4 h fasting (n = 10 per group). For ITT, insulin (0.75 U/kg) was i.p. injected and blood glucose levels were measured at different time points (0, 15, 30, 60, and 120min) after insulin injection [23]. Blood glucose levels were measured using a glucometer (Roche Accu-Chek Inform).

### Behavioral methods and procedures

#### Open field test

Open field test was conducted to test anxiety-related activities at the 24th week (n = 10 per group) [24]. The apparatus was 20 × 20 cm with 28 cm high wooden walls. The box floor was painted with white lines to form 16 equal squares with a colored box (8 × 5 × 3 cm) in the center of the area. Mice were individually placed in the left corner of a square, facing the walls and was permitted to explore the environment for 5 min *ad lib*. The following parameters were recorded: latency to first entry, peripheral distance, peripheral time, central distance, total number of squares crossed, the number of rearing, grooming and manure.

#### Elevated plus maze

Elevated plus maze was conducted to test anxiety-related activities one day after open field (n = 10 per group) [24]. The apparatus consisted of an x-shaped maze elevated 80 cm from the floor comprising two opposite enclosed arms (30 cm long, 5 cm wide, 15 cm high), two opposite open arms (30 cm long, 5 cm wide, without edges) and a central arena (5× 5 cm). Mice were placed individually in the central arena of the apparatus facing an open arm and was permitted to explore the environment for 5 min *ad lib*. Following parameters will be recorded and evaluated: the number of entries in open arms (4-paw criterion), time spent in open arms, open arm distance, the ratio of open/total arm entry, open/total arm distance and open/total arm time.

#### Morris water maze

Morris water maze (MWM) is a behavioral test in which rodents learn to find a platform hidden in the water. It is often used to test learning and memory performances [20]. It was started at the day after elevated plus maze (n = 10 per group) [24]. The entire test was completed in 7 days. The circular black water tank was 150 cm in diameter, 30 cm in height, with water 25 cm in depth and 24–26°C in temperature. A black escape platform (10 cm diameter, 24 cm high) was positioned in one of the four quadrants of the maze. In the first six days, mice were tested with 4 trials per day to find the submerged platform during spatial learning trials. On day 7, the platform was removed and spatial memory was test by spatial probe test. An automated tracking system was used to analysis the latency to find the platform, swim distance, swim velocity, the percentage of distance in the fifth zone and the time proportion in the 5th zone.

### Biochemical parameters

Serum samples were sent to the clinical laboratory of the Second Affiliated Hospital of Anhui Medical University for testing. Serum folate was measured by chemiluminescent immunoassay (Simens Immulite2000, UK) using folic acid assay kit (Simens). Total serum homocysteine was detected by colorimetric method (Beckaman AU5800, USA) using homocysteine assay kit (Leadman Biochemistry, Beijing). Serum insulin was detected by electrochemiluminescence immunoassay (Roche Cobase602, Germany) using insulin assay kit (Roche). Serum lipid parameters were detected by an automatic biochemical analyzer (Beckman AU5800, USA). Serum nonesterifed fatty acid was determined by colorimetric acyl-CoA synthetase and acyl-CoA oxidase-based methods. The nonesterifed fatty acid assay kit was purchased from Shanghai Kehua Bioengineering Institute (Shanghai, China). Serum total cholesterol was measured by the cholesterol oxidase method. Serum triglyceride was measured by standard enzymatic methods. Serum high density lipoprotein (HDL) cholesterol was measured by polyanion polymer/detergent HDL-C assay. Serum low density lipoprotein (LDL) cholesterol was determined by the homogeneous method. Assay kits of cholesterol, triglyceride, HDL and LDL were purchased from Beckman Coulter Inc. Serum very low density lipoprotein (VLDL) cholesterol was measured by the Friedewald formula (VLDL = TG × 0.2).

### Histology

Liver tissues were fixed overnight at 4°C in 4% paraformaldehyde. Samples were gradually dehydrated in ethanol, embedded in paraffin. and then sliced into 4 μm sections. Hematoxylin-eosin (H&E) staining was performed to evaluate hepatic lipid accumulation. Images were obtained by a microscopy (Olympus DX53, Japan).

### Phosphatidylcholine assay

Phosphatidylcholine level in liver tissues was measured by colorimetric using phosphatidylcholine assay kit (ab83377, from Abcam, Cambridge, UK). Liver tissues were washed with cold PBS, resuspended in the assay buffer provided by the kit, and homogenized with a Dounce homogenizer on ice. Thereafter, samples were centrifuged for 5 min at 4°C at 12,000 × g to exclude the insoluble material and collect the supernatant. The supernatant was incubated on a 96-well plate with the reaction mix for 30 min at room temperature and were protected from light. The colorimetric reaction was measured at 570 nm. Optical density was measured using a microplate reader. Then the concentration of phosphatidylcholine was estimated.

### Immunoblots

The tissues was homogenized in RIPA buffer containing complete-mini protease inhibitor and cleared by centrifugation. The supernatant was collected for immunoblotting. Protein was separated using SDS-PAGE and transferred onto a polyvinylidene fluoride membrane. The experiments were carried out as described [25]. The membranes were incubated for 2 hours with the following antibodies: Akt, p-Akt and β-actin was used as a loading control antibody. After washing in Dulbecco’s phosphate-buffered saline containing 0.05% Tween 20, the membranes were incubated with goat anti-rabbit IgG antibody for 2 hours. The membranes were washed in Dulbecco’s phosphate-buffered saline containing 0.05% Tween 20, followed by signal development using an enhanced chemiluminescence detection kit (Pierce Biotechnology, Rockford, IL, USA). After developing, the X-ray films were scanned and densitometry analyses were performed with NIH Image J software. Antibodies: anti-p-Akt (Ser473), Cell Signaling Technologies, Danvers, MA, USA, 4060s, rabbit monoclonal 1:2000; anti-Akt, Cell Signaling Technologies, 4691s, rabbit monoclonal 1:2000; anti-β-actin, Beijing Biosynthesis Biotechnology, Beijing, China, bsm-33036M, rat monoclonal 1:1000.

### Isolation of total RNA and real-time RT-PCR

Total RNA was extracted using TRI reagent (Invitrogen, Carlsbad, CA, USA). cDNA synthesis was performed as described [23]. Real-time RT-PCR was performed with a LightCycler 480 SYBR Green Imasterq PCR mix (Roche Diagnostics, Indianapolis, IN, USA) using gene-specific primers as listed in **S3 Table**. The amplification reactions were carried out on a LightCycler 480 instrument (Roche Diagnostics). The comparative cycle threshold method was used to determine the amount of target [26], normalized to an endogenous reference (18s) and relative to a calibrator using the LightCycler 480 software (version 1.5.0; Roche). All the RT-PCR experiments were performed in triplicate. The primers were synthesized by Shanghai Sangon Biological Engineering Technology and Service Company (Shanghai, China).

## Statistical analysis

Normally distributed data were expressed as mean ± means of standard error (SEM). The differences between two groups were analyzed using independent-samples *T*-Test. The test data from the IPGTT, ITT, MWM tasks, and body weight, were analyzed by Repeated Measures Analysis of Variance (rm-ANOVAs) using Fisher’s least-significant difference test for post hoc analysis. For immunoblotting, developed films were scanned and band intensities were analyzed using the public domain NIH Scion Image Program. All analyses were conducted by statistical software, SPSS 20.0 for Windows. *P* < 0.05 was considered statistically significant.

## Results

### Effects of CFD diet on serum folate and total homocysteine levels

Serum folate and total homocysteine levels were examined after a 25-week diet treatment. As expected, serum folate levels were approximately 5-fold lower in CFD diet-fed mice as compared with controls (57.59 ± 3.09 vs. 9.10 ± 0.89 nmol/L, *P*<0.001). Serum total homocysteine levels were increased in CFD diet-fed mice as compared with controls (14.43 ± 0.48 vs. 22.01 ± 2.57 μmol/L, *P* = 0.012).

### Effects of CFD diet on lipid metabolism in mice

**Fig 1:**
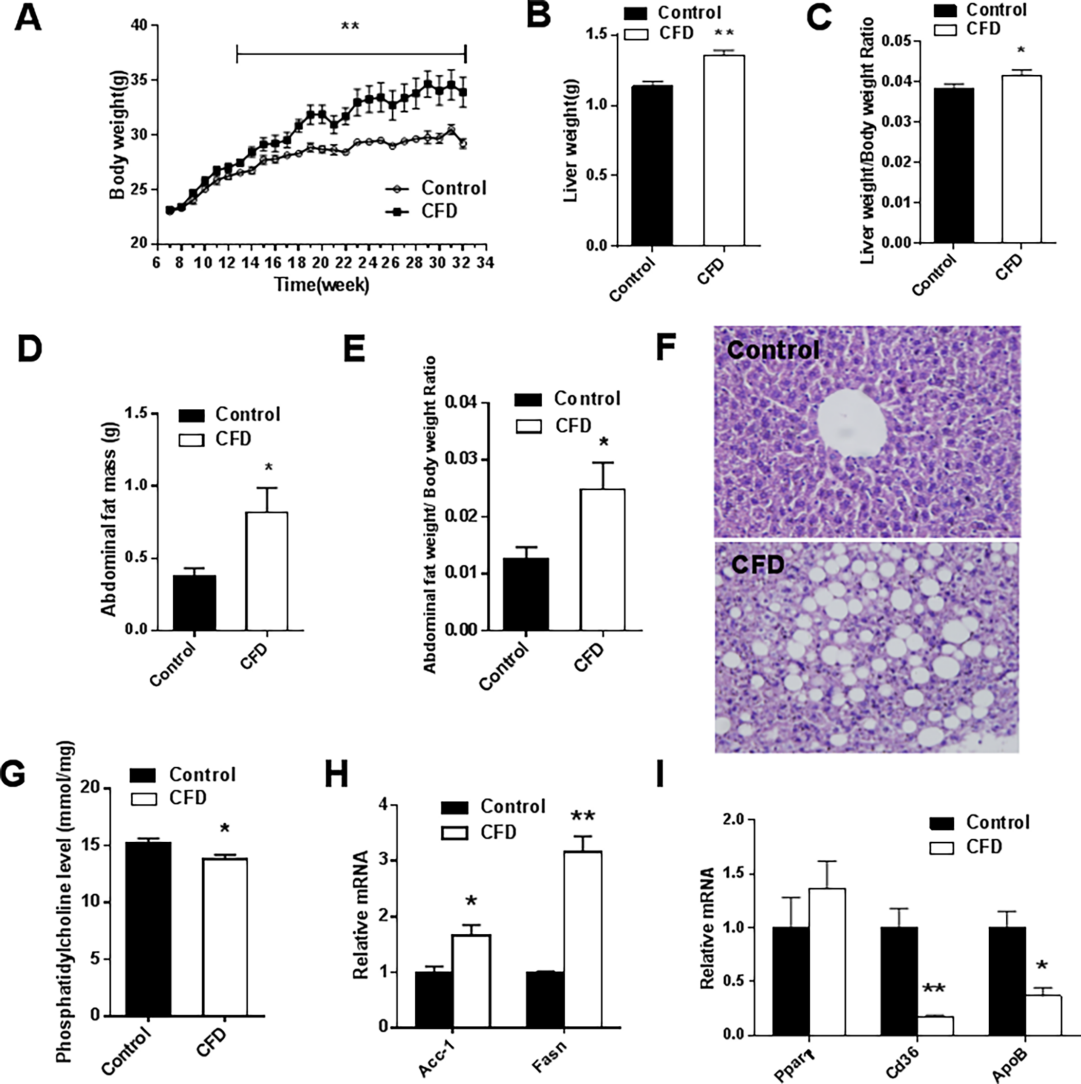
Effects of CFD diet on lipid metabolism in mice. ICR mice were fed with either a CFD or control diet for 25 weeks. Mice were weighed every week. (A) Body weight (n = 10 per group). (B) Liver weight. (C) Liver weight/body weight ratio (n = 7 per group). (D) Abdominal fat weight (n = 7 per group). (E) Abdominal fat weight/body weight ratio (n = 7 per group). (F) Liver sections were stained with hematoxylin and eosin (original magnification, ×200) (n = 3 per group). (G) Hepatic phosphatidylcholine level (n = 7 per group). (H) Hepatic lipid-related genes were examined by real-time RT-PCR (n = 3 per group). Hepatic fatty acid synthesis genes, *Acc1*, *Fasn*. (I) Hepatic lipid transport genes, *Pparγ*, *Cd36*, *ApoB*. All data were expressed as mean ± SEM. *P < 0.05, **P < 0.01. Abbreviation: *Acc1*, acetylCoenzyme A carboxylase 1; *Fasn*, fatty acid synthase; *Pparγ*, peroxisome proliferator-activated receptor; *Cd36*, cluster of differentiation; *ApoB*, apolipoprotein B.

The effect of CFD diet on body weight was evaluated every week. After a 13-week diet treatment, the body weight was significantly increased in the CFD group as compared with controls **(Fig 1A)**. As shown in **Fig 1B**, the absolute liver weight was markedly increased in the CFD group (*P*<0.001). Accordingly, the liver/body weight ratio was significantly increased in mice exposed to CFD diet (*P* = 0.046) **(Fig 1C)**. Moreover, liver sections stained with H&E showed increased fat vacuoles, and also increased cell size, which related to hepatic lipid accumulation in the CFD group **(Fig 1F)**. The effects of CFD diet on abdominal fat mass were then analyzed. Abdominal fat mass was significantly increased in mice fed with CFD diet (*P* = 0.016). As revealed in **(Fig 1D)**. Correspondingly, the abdominal fat weight/body weight ratio was markedly elevated in the CFD group (*P* = 0.019) **(Fig 1E)**. In addition, serum triglyceride (*P*<0.001) and VLDL cholesterol levels (*P*<0.001) were significantly increased in the CFD group compared with controls **(Table 1)**.

**Table 1:** Circulating parameters of lipids and glucose metabolism.

	Control (n = 6)	CFD (n = 6)	*P* value
Fasting blood glucose (mmol/L)	5.55±0.17	6.67±0.41*	0.022
Serum insulin (pmol/L)	7.60±0.75	4.02±0.71**	0.005
Serum triglycerides (mmol/L)	0.66±0.04	1.51±0.14**	0.004
Serum nonesterifed fatty acid (mmol/L)	1.25±0.16	1.47±0.24	0.189
Serum total cholesterol (mmol/L)	3.34±0.34	2.97±0.31	0.215
Serum HDL cholesterol (mmol/L)	2.55±0.26	2.32±0.22	0.238
Serum LDL cholesterol (mmol/L)	0.75±0.09	0.62±0.13	0.231
Serum VLDL cholesterol (mmol/L)	0.13±0.01	0.30±0.03**	0.005

*P < 0.05

**P < 0.01. Abbreviation: HDL, high density lipoprotein; LDL, low density lipoprotein; VLDL, very low density lipoprotein.

To explore the mechanism of hepatic lipid accumulation in the CFD group, phosphatidylcholine level and genes for fatty acid synthesis and lipid transport in the liver tissues were then analyzed. Phosphatidylcholine level was significantly reduced in CFD group (*P* = 0.013) **(Fig 1G)**. *Fasn* (*P*<0.001) and *Acc1 *(*P* = 0.022) were significantly upregulated in the liver tissues of CFD diet-fed mice **(Fig 1H)**. The expression of *Cd36* and *ApoB* mRNA were significantly downregulated in the CFD group (*P* = 0.005; *P* = 0.010, respectively) **(Fig 1I)**.

### Effects of CFD diet on glucose metabolism

**Fig 2:**
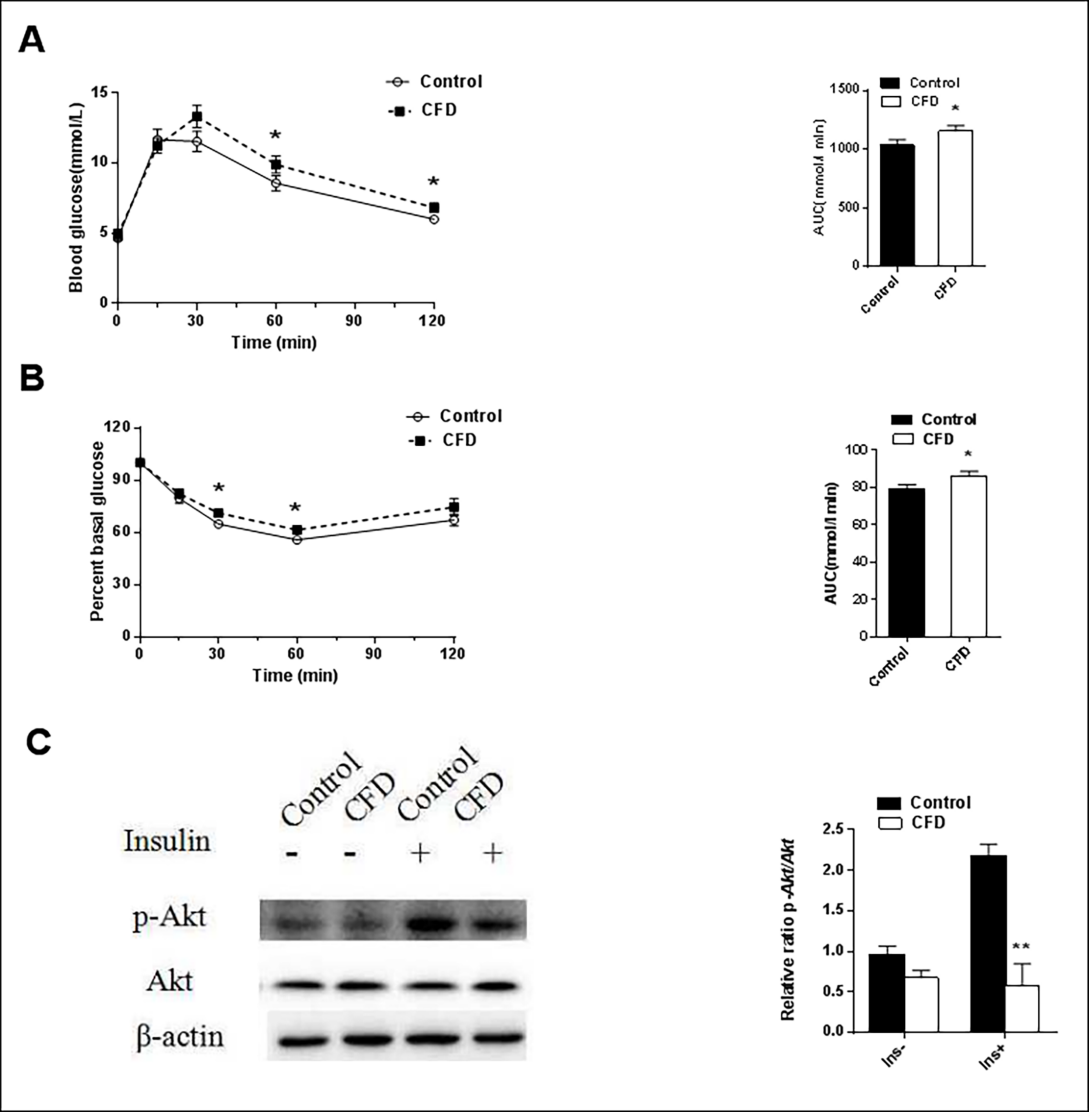
Effects of CFD on glucose metabolism in mice. Mice were fed with a CFD or control diet. (A) Blood glucose and area under the glucose curve during IPGTT after a 16-week diet treatment (n = 10 per group). (B) Percent basal glucose levels and the inverse integrated area under the glucose curve during ITT after a 17-week diet treatment (n = 10 per group). (C) Hepatic p-Akt/Akt protein ratio (n = 3 per group). Data were expressed as mean ± SEM. *P <0.05, **P < 0.01. Abbreviation: IPGTT, intraperitoneal glucose tolerance test; ITT, intraperitoneal insulin tolerance test; p, phosphorylated.

The effects of CFD on glucose tolerance and insulin tolerance were analyzed using IPGTT and ITT at the 16th and 17th week after diet treatment. As shown in **Fig 2A**, CFD diet-fed mice had significantly higher blood glucose levels at 0, 60, 120 minutes following glucose injection as compared with control mice (*P* = 0.043; *P* = 0.044 and *P* = 0.049, respectively). As shown in **Fig 2B**, CFD diet-fed mice had significantly higher percent basal glucose levels at 30 and 60 minutes following insulin injection as compared with control mice (*P* = 0.035; *P* = 0.047, respectively). Compared with control mice, CFD diet-fed mice presented a significant elevation of glucose area under the curve (AUC) during the IPGTT and inverse AUC during ITT (*P* = 0.015 and *P* = 0.046, respectively) **(Fig 2A and 2B)**. The glucose levels in ITT were showed in S1 Fig. The tests showed CFD induced glucose intolerance and insulin resistance.

As shown in **Table 1**, the fasting blood glucose levels were significantly higher in the CFD group as compared with control group (*P* = 0.022). Of interest, there was a significant reduction in serum insulin levels in CFD group as compared with control group (*P* = 0.005). Furthermore, the hepatic p-Akt levels were increased in response to insulin challenge. However, the hepatic p-Akt/Akt ratio was significantly decreased in CFD-diet fed mice (*P* = 0.003) **(Fig 2C)**. The results showed CFD induced hyperglycemia and inhibited hepatic insulin pathway.

### Effects of CFD diet on cognitive behaviors

#### Open filed test

The effects of CFD on anxiety-related activities were detected by open field test and elevated plus maze in the 24th week after diet treatment. In the open-field test, the peripheral time (*P* = 0.032) and the number of grooming (*P* = 0.020) were significantly increased in CFD diet-fed mice, whereas CFD diet had little effect on the latency to the first grid crossing (*P* = 0.128), central distance (*P* = 0.184) and peripheral distance (*P* = 0.375). In addition, no significant difference was observed in the number of squares crossed (*P* = 0.326), rearing (*P* = 0.260) and manure (*P* = 0.287) **(Table 2)**.

**Table 2:** The performance in the open field test.

	Control (n = 10)	CFD (n = 10)	*P* value
Latency (s)	35.12±8.75	55.29±14.83	0.128
Peripheral distance (m)	38.99±3.33	40.43±2.94	0.375
Peripheral Time (s)	275.91±4.54	286.33±2.66*	0.032
Central distance (m)	5.24±0.91	3.99±0.99	0.184
Squares crossed	28.10±3.97	25.10±5.22	0.326
Grooming	10.20±1.60	15.80±1.96*	0.020
Rearing	23.50±3.06	26.80±4.00	0.260
Manure	2.60±0.62	2.20±0.33	0.287

Data were expressed as mean ± SEM.

*P < 0.05 as compared with controls.

#### Elevated plus maze

In the elevated plus maze, the distance in the open arms (*P* = 0.008), open/total distance ratio (*P* = 0.008) and open/total arm entries ratio (*P*<0.001) were significantly decreased in CFD diet-fed mice. However, there was no significant difference in number of entries (*P* = 0.207) and time spent in open arms (*P* = 0.161). Moreover, open/total arm time ratio was not affected by CFD diet (*P* = 0.089) **(Table 3)**. These results showed that CFD diet induced anxiety-related activities.

**Table 3:** The performance in elevated plus maze.

	Control (n = 10)	CFD (n = 10)	*P* value
Open arm entries	16.80±1.95	14.72±1.55	0.207
Open arm distance (m)	0.20±0.03	0.11±0.02**	0.008
Open arm time (s)	101.60±14.93	82.42±11.54	0.161
Open/total arm entries	0.47±0.15	0.16±0.02**	<0.001
Open/total arm distance	0.25±0.04	0.13±0.02**	0.008
Open/total arm time	0.47±0.06	0.36±0.04	0.089

Data were expressed as mean ± SEM.

*P < 0.05

**P < 0.01 as compared with controls.

#### Morris water maze

**Fig 3:**
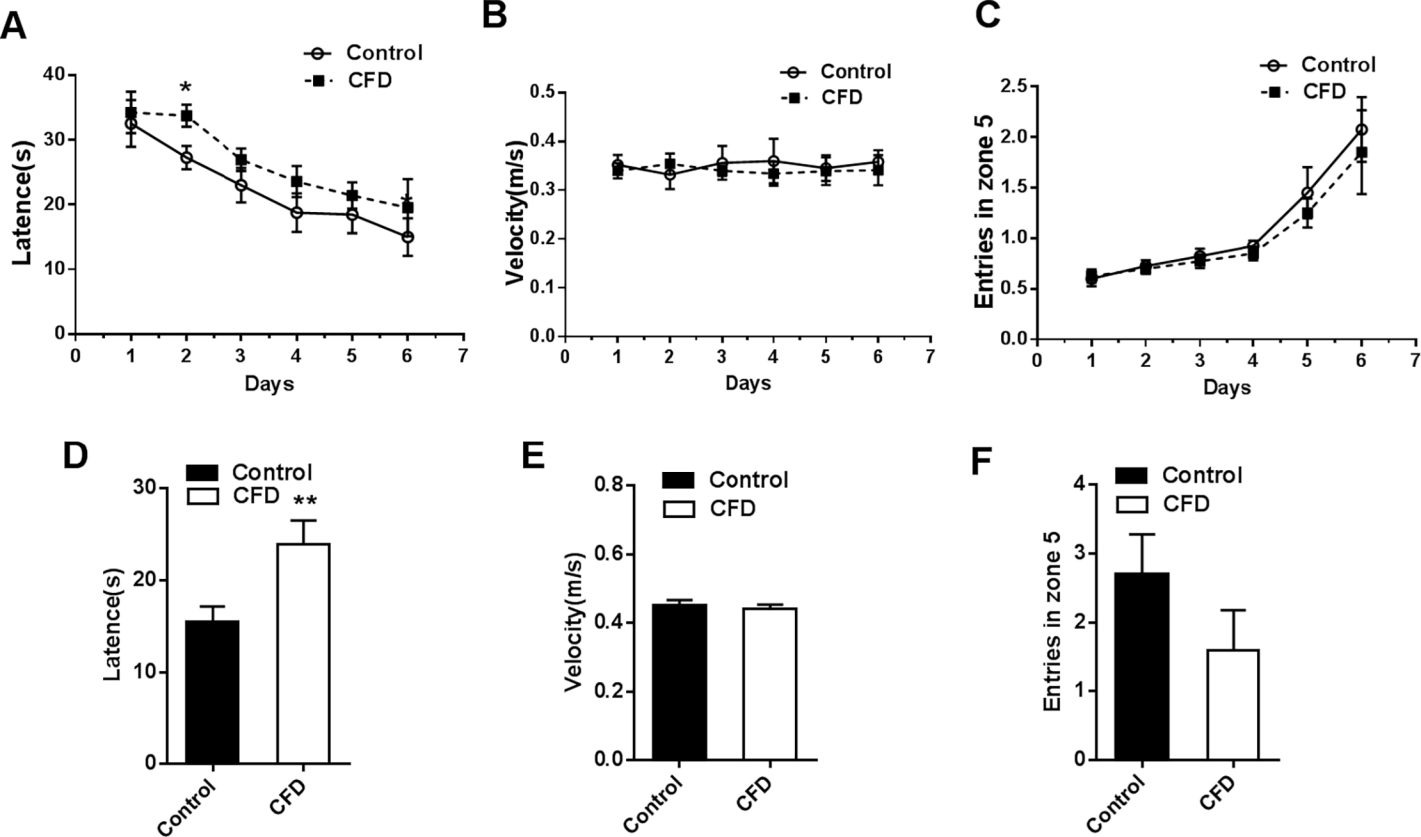
The effect of CFD diet on learning and memory performance. MWM was conducted to test learning and memory performances after a 24-week diet treatment. (A-C) Learning performance was examined on the first six days. (A) The latency to find the platform. (B) Swim velocity. (C) Entries in zone 5. (D-E) Memory performance was assessed on day 7. (D) The latency to find the platform. (E) Swim velocity. (F) Entries in zone 5. All data were expressed as mean ± SEM (n = 10 per goup). *P < 0.05, **P < 0.01. Abbreviation: MWM, Morris water maze.

The effects of folate deficiency on learning and memory performance were detected by MWM after a 24-week diet intervention. In MWM, the learning performances in the first six days were analyzed. The latency to platform shortened progressively daily for all mice combined (*F* = 10.906, *P*<0.001) **(Fig 3A)**. The latency to find the platform (*F* = 4.640, *P* = 0.045) was longer in the CFD group than control group **(Fig 3A)**. The results showed that there was no significant difference in swim velocity between two groups, (*F* = 0.149, *P* = 0.704), and days (*F* = 0.030, *P* = 1.000) **(Fig 3B)**. Moreover, the number of entries in zone 5 was increased daily in both two groups (*F* = 18.174, *P*<0.001) **(Fig 3C)**. However, there was no significant difference in the number of entries in zone 5 between two groups (*F* = 0.479, *P* = 498) **(Fig 3C)**. Spatial memory was then assessed on day 7. As shown in **Fig 3D,** the latency in the CFD group was significantly increased (*P* = 0.007). However, there was no significant difference on swim velocity (*P* = 0.348) and entries in zone 5 (*P* = 0.098) between the two groups **(Fig 3E and 3F).** These results suggested that folate deficiency impairs the ability of learning and memory in CFD mice.

## Discussion

Previous studies have shown lower folate levels and higher homocysteine levels in patients with T2D as compared with non-diabetic subjects [27–29]. A cohort study showed that dietary folate intake was inversely associated with risk of T2D for women, not for men [30]. Moreover, T2D patients with cognitive decline predispose to have lower folate levels compared with T2D patients without cognitive decline [5]. However, whether folate deficiency could cause glucose metabolism disorders and subsequently impaire cognitive function in female is still unknown. Therefore, the present study explored the effects of CFD on glucose and lipid metabolism and cognitive function in female ICR mice. Interestingly, our data showed that CFD induced obesity, insulin resistance, and subsequent cognitive dysfunction.

The present study showed that obesity occurred after treatment of folate deficient diet in mice. CFD also led to lipid metabolism disorders. It was recognized that folate and phosphatidylcholine metabolism are inter-related. An animal study showed that folate deficiency decreased flux through phosphatidylethanolamine N-methyltransferase, an enzyme that synthesizes phosphatidylcholine via the methylation of phosphatidylethanolamine [31]. Phosphatidylcholine is a major component of VLDL, which transport triglycerides out of liver [32]. Therefore, decreased phosphatidylcholine levels would lead to the reduction of VLDL in the liver tissues and the accumulation of hepatic triglycerides [33]. The present study showed increased serum triglycerides and VLDL levels. In cystathionine beta-synthase-deficient mice, an animal model for hyperhomocysteinemia, elevated serum homocysteine levels inhibited intravascular VLDL lipolysis, resulting in elevated serum VLDL levels [34]. Previous studies demonstrated that lower phosphatidylcholine promoted *Srebp1* proteolytic maturation and its target gene expression, including *Acc1* and *Fasn*, two rate-limiting enzymes for fatty acid synthesis [35–37]. In agreement with these results, Yu X et al. showed that folate supplement decreased the expression of *Fasn* mRNA [38]. However, CFD decreased the fatty acid transporter *Cd36* mRNA [39]. These results suggested that CFD increased hepatic fatty acid synthesis whereas limited lipid transporting out of the liver. Moreover, histopathology showed that hepatic lipid accumulation, a known determinant of insulin resistance, was observed in the CFD group. These results suggested that CFD induced lipid metabolism disorders, which may be related to insulin resistance.

CFD diet-fed mice showed glucose intolerance and insulin resistance. Moreover, CFD significantly decreased serum insulin levels. A vitro study demonstrated that folate deficiency condition could trigger oxidative-nitrosative stress, and subsequent endoplasmic reticulum stress in the insulin-producing pancreatic islets RINm5F cells [40]. These events resulted in apoptosis of RINm5F cells, as well as impairment of the biosynthesis and the secretion of insulin. Insulin signaling plays an important role in hepatic glucose metabolism. Insulin binds to insulin receptors and then triggers the activation of phosphoinositide 3-kinase, which in turn triggers the activation of Akt kinase. Lower phosphorylation of Akt can decrease glycogen content by inhibiting glycogen synthase through the activation (inhibition of phosphorylation) of GSK3β [41]. The present study showed hyperhomocysteinemia in the CFD group. Elevated homocysteine levels led to increased levels of its metabolite homocysteine thiolactone [42]. Homocysteine thiolactone could inhibit insulin receptor tyrosine kinase activity, which resulted in decreased phosphatidylinositol 3-kinase activity, and attenuate the phosphorylation of Akt [43–44]. In conclusion, CFD caused insulin resistance in mice.

Several epidemiological reports demonstrated that T2D was a strong predictor for anxiety and memory deficits in older adults [45–46]. The Whitehall II cohort study of 5653 participants showed that midlife people with T2D had a 45% faster decline in memory, a 24% faster decline in the global cognitive score in early old age [46]. An animal experiment showed that escape latency of type 2 diabetic mice was significantly longer in the MWM test [47]. In the *APP/PS1* transgenic mice model of Alzheimer disease, glucose tolerance and insulin sensitivity were impaired 6–7 months prior to amyloid plaque pathogenesis and cognitive dysfunction [48]. In the present study, the peripheral time and the number of grooming were significantly increased in the CFD group in the open-field test. In the elevated plus maze, percentage of open/total arm entries was significantly decreased in the CFD group compared with controls. In MWM, the latency to find the platform was longer in the CFD group than control group. Glucose is the main brain energy substrate which can alter glutamate-glutamine cycle homeostasis in hippocampus. Glutamate homeostasis can affect the neuron and neighboring astrocytes. The dysfunction in hippocampus could cause behavior deficits [49]. In conclusion, this study showed that CFD diet induced anxiety related activities, and impairment in spatial learning and memory which may related with insulin resistance.

The present study had several limitations. First, this study focused on the effects of CFD diet on glucose and lipid metabolism and cognitive function in female mice, but it could not explore gender difference. Second, succinylsulfathiazole intake was reported to modify the intestinal microbiome, including reduction of *Lactobacillus* and *Coliform* and upregulation of *Enterococci* and *Yeastlike* organisms in Sprague-Dawley rats [50]. Changes in intestinal microbiome have been closely associated with glucose and lipid metabolism. However, the causality has not been proven yet [51]. In addition, administration of *Enterococcus faecium* WEFA23 can improve key markers of metabolic syndrome, including obesity, hyperlipidemia, hyperglycemia, and insulin resistance [52]. Probiotic *Lactobacillus gasseri* SBT2055 (LG2055) showed beneficial influence on metabolic disorders [53]. Therefore, it is uncertain what effect succinylsulfathiazole-induced microbiome modification had on glucose and lipid metabolism in this study. Further research is required to clarify the potential influence of the altered microbiome.

In summary, the present study investigated the effects of CFD on glucose and lipid metabolism and cognitive function in female ICR mice. CFD induced obesity, lipid metabolism disorders, insulin resistance and inhibited the insulin signaling. Moreover, CFD increased anxiety related activities and impaired the ability of spatial learning and memory. The present study provided the evidence that CFD could cause glucolipid metabolism disorders, and subsequent cognitive dysfunction, which might be related to CFD-induced insulin resistance. Future studies are needed to investigate the effect of folic acid supplementation on lipid and glucose metabolism.

## Supporting information

S1 Table The composition in diets. (DOC)

S2 Table Percentage distribution of calories. (DOC)

S3 Table Oligonucleotide sequence of primers for real-time RT-PCR. (DOC)

S1 Fig The glucose levels in ITT. Mice were fed with a CFD or control diet. glucose levels and AUC in the ITT after a 17-week diet treatment (n = 10 per group). Data were expressed as mean ± SEM. *P <0.05, **P < 0.01.
(ZIP)

## Abbreviations

CFD: chronic folate deficiency
HDL: high density lipoprotein
IPGTT: intraperitoneal glucose tolerance tests
ITT: intraperitoneal insulin tolerance tests
LDL: low density lipoprotein
MWM: Morris water maze
T2D: Type 2 diabetes
TG: triglyceride
VLDL: very low density lipoprotein
