# Supplementary material for: Chronic folate deficiency induces glucose and lipid metabolism disorders and subsequent cognitive dysfunction in mice
Source: PLoS One. 2018 Aug 28;13(8):e0202910. doi: 10.1371/journal.pone.0202910 (PMC6112663; doi:10.1371/journal.pone.0202910)
Supplement: S1 Table — (DOC) [file pone.0202910.s001.doc]

**S1 Table. The composition in diets**

| Ingredient (g/kg) | Control   | **g/kg** | | --- | | CFD |
| --- | --- | --- | --- |
| L-amino acid mix | 183.38 | 183.38 |
| Starch | 404.14 | 404.14 |
| Maltodextrin | 130.34 | 130.34 |
| Sucrose | 98.75 | 98.75 |
| Soybean oil | 69.12 | 69.12 |
| Cellulose | 49.37 | 49.37 |
| Mineral mix | 34.56 | 34.56 |
| Vitamin mix | 9.87 | 9.87 |
| Choline bitartrate | 2.47 | 2.47 |
| Sodium bicarbonate | 7.60 | 7.60 |
| Succinylsulfathiazole | 0.00 | 10.37 |
| TBHQ | 0.01 | 0.01 |
| Folate | 0.002 | 0 |
