# Supplementary material for: Chronic folate deficiency induces glucose and lipid metabolism disorders and subsequent cognitive dysfunction in mice
Source: PLoS One. 2018 Aug 28;13(8):e0202910. doi: 10.1371/journal.pone.0202910 (PMC6112663; doi:10.1371/journal.pone.0202910)
Supplement: S2 Table — (DOC) [file pone.0202910.s002.doc]

**S2 Table. Percentage distribution of calories**

|  | Control | CFD |
| --- | --- | --- |
| Sugar (%energy) | 19.4 | 19.4 |
| Protein (%energy) | 63.9 | 63.9 |
| Fat (%energy) | 16.7 | 16.7 |
| Total energy (kcal/g) | 3.7 | 3.7 |
