# Supplementary material for: Chronic folate deficiency induces glucose and lipid metabolism disorders and subsequent cognitive dysfunction in mice
Source: PLoS One. 2018 Aug 28;13(8):e0202910. doi: 10.1371/journal.pone.0202910 (PMC6112663; doi:10.1371/journal.pone.0202910)
Supplement: S3 Table — (DOC) [file pone.0202910.s003.doc]

**S3 Table. Oligonucleotide sequence of primers for real-time RT-PCR**

| Genes | Forward (5’-3’) | Reverse (5’-3’) |
| --- | --- | --- |
| 18s | CGGCTACCACATCCAAGGAA | GCTGGAATTACCGCGGCT |
| Acc1 | CCGTTGGCCAAAACTCTGGAGCTA A | GAGCTGACGGAGGCTGGTGACA |
| Fasn | CGCTCGGCTCGATGGCTCAG | CCAGCACCACGGCAT GCTCA |
| Pparγ | GGGCTGAGGAGAAGTCACAC | TCAGTGGTTCACCGCTTCTT |
| Cd36 | CACAGCTGCCTTCTGAAATGTGTGG | TTTCTACGTGGCCCGGTTCTAATTC |
| ApoB | AGAGGCCAGTCAAGCTGT TC | GCGTTGGAGTAAGCTCCTGT |
