# Supplementary material for: Chronic folate deficiency induces glucose and lipid metabolism disorders and subsequent cognitive dysfunction in mice
Source: PLoS One. 2018 Aug 28;13(8):e0202910. doi: 10.1371/journal.pone.0202910 (PMC6112663; doi:10.1371/journal.pone.0202910)
Supplement: S1 Fig — Mice were fed with a CFD or control diet. glucose levels and AUC in the ITT after a 17-week diet treatment (n = 10 per group). Data were expressed as mean ± SEM. *P <0.05, **P < 0.01. (ZIP) [file pone.0202910.s004.zip › S1 Fig. file/S1_Fig.doc]

**S1 Fig. The glucose levels in ITT.** Mice were fed with a CFD or control diet. glucose levels and AUC in the ITT after a 17-week diet treatment (n=10 per group). Data were expressed as mean ± SEM. **P* <0.05, ***P* < 0.01.
